# Quieting "Food Noise": How GLP-1s and Mindfulness Rewire the Default Mode Network (DMN) and Reward Circuits

**DOI:** 10.7759/cureus.100818

**Published:** 2026-01-05

**Authors:** Geoff Cook

**Affiliations:** 1 Nutrition, Noom, Princeton, USA; 2 Nutrition, Friedman School of Nutrition, Tufts University, Boston, USA

**Keywords:** default mode network, dmn, executive function, food noise, glp-1, glp-1 receptor agonists, mindfulness, obesity, prospection, semaglutide

## Abstract

This narrative review synthesizes evidence identified through targeted searches of PubMed, Scopus, and Web of Science (2010-2025), with a focused selection of human and mechanistic studies examining GLP-1 receptor agonists (GLP-1s), food cue reactivity, prospective cognition, mindfulness-based interventions, and neural network dynamics relevant to 'food noise' and the default mode network (DMN). Food noise is conceptualized here as a form of maladaptive prospection: a faulty way of thinking about the future, characterized by repetitive, cue-driven mental simulation of short-term reward at the expense of long-term goals. Existing neuroimaging and behavioral data suggest that GLP-1s may influence neural systems underlying cue salience and reward anticipation, with several reports indicating reduced food-related intrusive thoughts. Although these findings are preliminary, some mechanistic models posit that GLP-1s could attenuate DMN activity associated with food-related rumination, potentially altering the cognitive context in which eating decisions occur. Patient reports of improved focus, reduced cravings, or greater ease in health-related planning are noted in the literature, but causal links to specific behavioral outcomes remain unestablished. This paper advances a testable hypothesis: reductions in food noise may shift the balance of activity among DMN, salience, and executive networks in ways that support more adaptive forms of prospection. However, current evidence is limited, and the proposed mechanisms and behavioral implications require empirical testing. Further research using direct measures of food noise, longitudinal neuroimaging, and controlled behavioral studies is needed to clarify mechanisms and determine their broader relevance for health and self-regulation.

## Introduction and background

Food noise is defined as "persistent thoughts about food that are perceived by the individual as unwanted and/or dysphoric and may cause harm to the individual, including social, mental, or physical problems" [1]. Food noise is best understood not as a sensation of hunger but as a cognitive phenomenon, a form of maladaptive prospection in which the brain repeatedly simulates short-term reward scenarios in conflict with long-term goals.

Individuals attempting weight loss frequently describe intrusive, cue-triggered mental preoccupation with food even in the absence of physiological need. These thoughts are repetitive, prospective, vivid, and action-biasing.

To appreciate why this occurs, it is helpful to understand how appetite is regulated. Hunger is governed not only by the stomach or calorie needs but by a complex gut-brain communication network involving hormones such as GLP-1. GLP-1 is released in the gut after eating and travels through the bloodstream and vagus nerve to the brainstem and hypothalamus, where it slows gastric emptying, enhances satiety, and modulates reward pathways. Under typical circumstances, this system calibrates eating behavior to physiological need. But in environments saturated with calorie-dense cues, cognitive simulations about food can override biological signals, creating a gap between metabolic need and mental preoccupation.

In this framing, food noise is less an issue of physiological need and more an issue of cognitive load. This reframing matters because GLP-1s consistently reduce food noise in patients who describe the effect in cognitive rather than metabolic terms.

It is notable that obesity and depression often occur together, and both conditions show dysregulation in the default mode network (DMN) activity. This overlap hints that persistent intrusive food thoughts could share neural roots with the kind of rumination seen in mood disorders. "Food noise" can be understood as a byproduct of the DMN's tendency to wander and simulate rewards, which becomes problematic when it fixates on eating.

Now consider two very different approaches that appear to quiet this food-related chatter: GLP-1s and mindfulness meditation. GLP-1s are medications originally developed for type 2 diabetes that are now revolutionizing obesity treatment. Patients on drugs like semaglutide often report an uncanny mental silence regarding food, a noticeable drop in food noise.

Furthermore, GLP-1s increase liking and reduce wanting for food based on functional magnetic resonance imaging (fMRI) scans [2], a remarkable result that is reminiscent of a mindfulness practice and one that suggests research is needed into whether a GLP-1 increases mindful eating behaviors based on validated questionnaires, like the Mind-Eat scale [3].

In parallel, mindfulness practices operate through attention rather than through physiological mechanisms: they train individuals to stabilize awareness, observe cravings without acting on them, and disrupt habitual cue-response loops. How could interventions as disparate as a gut-hormone therapy and an ancient form of mental training both address the same problem? Research is needed to understand the linkage between GLP-1s and mindful eating.

This review suggests that food noise represents maladaptive prospection induced by DMN dysregulation; both GLP-1s and mindfulness reduce this dysregulation through distinct but convergent neural pathways. GLP-1s act on the body and brain chemistry to curb appetite and reward, while mindfulness acts on the mind's habits to curb rumination and reactivity. Both end up helping people eat less and feel more in control. This review examines five interconnected themes at the intersection of physiology and psychology, using two illustrative studies per theme to anchor the discussion.

First, this review explores the DMN and "food noise": how the DMN underpins mind-wandering and how food noise can be seen as a DMN-driven loop of intrusive, future-oriented thoughts about eating.

Second, this review discusses GLP-1s and the DMN: how GLP-1s influence the brain's resting-state networks, including the DMN, and possibly dampen background chatter about food.

Third, this review examines GLP-1 and food reward: how GLP-1s alter the brain's reward pathways and responses to food cues, reducing cravings and the drive to seek high-calorie rewards.

Fourth, this review discusses GLP-1's cognitive effects and role in addiction: evidence that GLP-1s may have far-reaching effects on other reward-seeking behaviors, such as alcohol use, and even on cognitive health, suggesting their impact extends beyond appetite and weight loss.

Fifth, this review explores mindfulness, rumination, and the DMN: how mindfulness meditation quiets the DMN and reduces maladaptive self-referential thinking, including food-related rumination, offering a non-pharmacological route to similar benefits.

After exploring these themes, this review integrates insights from GLP-1 and mindfulness, considering how each approach amplifies satiety signals, GLP-1 by increasing the physiological fullness signal and mindfulness by increasing attention to that signal, to help tame cravings.

This review also discusses how these approaches align with a model of emotion that uses the dimensions of valence (pleasure) and arousal (intensity of motivation). In particular, this review considers whether GLP-1s reduce the positive valence (the hedonic appeal) and reduce the high arousal (the urge) of food cues and how mindful eating practices might mediate those effects. Together, the findings shed light on how to turn down the "volume" on food noise, whether through pharmacology, mental training, or a combination of both.

At first glance, mindfulness training and GLP-1s appear to sit at opposite ends of the spectrum: one cultivates voluntary self-regulation, the other delivers a pharmacologic intervention. An open research question is whether GLP-1s, independent of any formal mindfulness practice, produce cognitive effects that resemble components of mindful eating. A related question is whether GLP-1s, when combined with a mindfulness practice, facilitate the adoption and maintenance of mindfulness more easily than training alone, and whether or not a combined program of GLP-1 and mindfulness yields greater or more sustained weight loss post-GLP-1 removal than either a pharmacotherapy program or a mindfulness program alone.

## Review

The DMN and "food noise"

Food noise is best understood as a DMN-driven form of maladaptive prospection.

The DMN is basically the brain's background setting, the network that takes over when we are not engaged in a specific task. It drives internally oriented thought, including mind-wandering, daydreaming, envisioning future plans, and reflecting on our lives. A landmark study by Killingsworth and Gilbert (2010) underscored how often our minds are in this mode [4]. Using a smartphone app to sample people's thoughts in real time, they found that minds were wandering, not on a task at hand, about 47% of the time. Interestingly, people reported being less happy when their minds were wandering than when they were focused on what they were doing. In other words, a wandering mind is common - and often unpleasant.

What kinds of thoughts fill our heads when our minds wander? A substantial body of research suggests that much of it is oriented toward the future. For instance, Stawarczyk et al. (2011) used experience sampling to analyze the content of people's off-task thoughts [5]. They found that the majority of spontaneous thoughts involved anticipating upcoming events, planning or worrying about events that had not yet occurred, or imagining hypothetical scenarios. In short, the default mode often operates as a mental simulator, engaging whenever attention drifts and running simulations of what is next on the agenda, what could go wrong, or what is already wrong and needs to be fixed.

This forward-looking tendency can become problematic if it turns into repetitive worry or cravings. Stawarczyk et al. observed that future-oriented mind-wandering episodes often have a ruminative flavor, characterized by loops of anticipation or anxiety. The concept of "food noise" fits into this picture as a specific case of the DMN being co-opted by a particular reward: food. When a person frequently overeats or is preoccupied with dieting, the DMN might latch onto food as its go-to topic during idle moments.

Hayashi et al. (2023) describe food-related intrusive thoughts (FRITs) as what happens when food cues - whether internal, like feeling a bit hungry, or external, like seeing a fast-food sign - trigger the DMN into a craving loop [6]. They propose a model in which a cue sparks cognitive reactivity, a craving thought, which then loops via attention networks and the DMN, leading to an outcome, like snacking on something unhealthy. This can occur even if you are not actually hungry; it is a mentally generated craving. Hayashi et al. note that GLP-1s have been reported to quiet this process, hinting at a link between these drugs and reduced DMN-driven rumination [6].

The need to measure food noise more formally led Diktas et al. (2025) to create the Food Noise Questionnaire (FNQ) [7]. In their study with adults who have obesity, higher FNQ scores - meaning more frequent and intrusive food thoughts - were associated with a greater tendency to overeat. This provides empirical support that "food noise" is not just a catchy phrase but a real cognitive phenomenon that varies between individuals and correlates with eating behavior.

**Figure 1:**
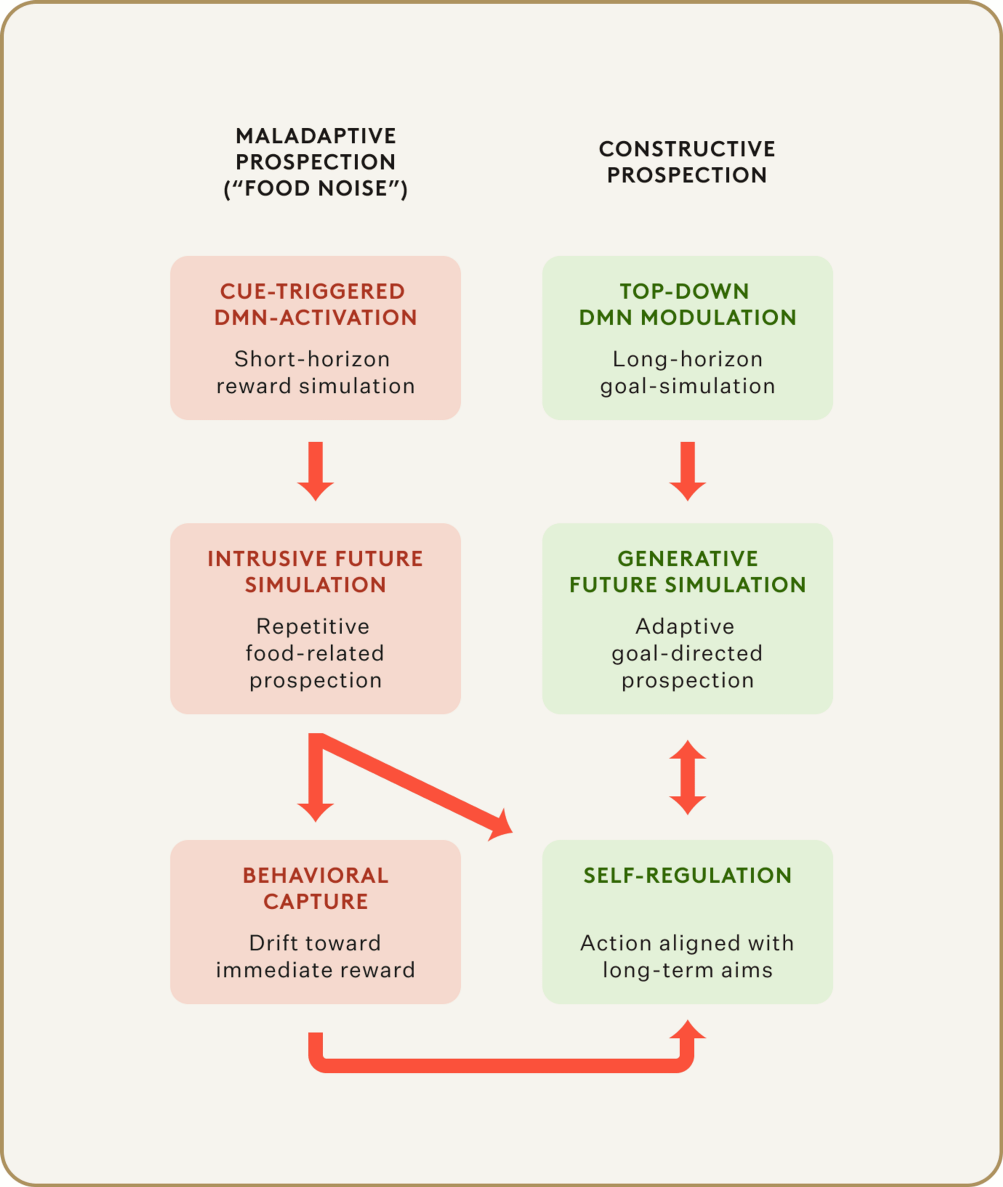
Maladaptive vs. Constructive Prospection Permission was obtained from the original publisher [9].

One can draw an analogy to mood disorders. Some researchers argue that depression involves "maladaptive prospection," where the DMN gets stuck generating negative, self-defeating thoughts about the future, feeding into low mood (Figure 1) [8,9]. Similarly, we might think of chronic overeating as involving maladaptive prospection about food - the DMN continually envisioning immediate food rewards and drowning out other thoughts. Notably, obesity is associated with a higher risk of depression, and neuroimaging studies show DMN abnormalities in both. While not causation, it is suggestive that a chronically overactive DMN could underlie both depressive rumination and obsessive food thoughts.

In summary, people spend substantial time in a default-mode state, during which the mind often wanders into the future. It is easy for such concerns to center on food, especially for individuals predisposed to cravings. "Food noise" can therefore be viewed as a manifestation of the DMN's chatter. Such a framing is useful because it links the subjective experience of "food noise" to a well-studied brain network. If food noise lives in the DMN, then perhaps strategies that quiet the DMN could help quiet food noise.

GLP-1s and the DMN

GLP-1s are best known for their effects on appetite; they slow gastric emptying, so you feel full longer, enhance insulin secretion, and send strong "full" signals from the gut to the brain. But what do they do to the brain's own activity patterns, especially when the brain is at rest? Emerging evidence suggests that GLP-1 analogs can modulate important brain networks, including the DMN. This is intriguing because if food noise is indeed rooted in DMN activity, a drug that alters the DMN might reduce that constant internal chatter about food.

A proof-of-concept study by van Duinkerken et al. (2021) examined this idea in women with obesity who underwent Roux-en-Y gastric bypass (RYGB) surgery [10]. RYGB is known to dramatically raise GLP-1 levels after meals, which is one reason patients feel less hungry post-surgery. The researchers performed resting-state fMRI scans on nine patients before and after surgery. To directly test the GLP-1's effect, they also infused a GLP-1 receptor blocker (exendin(9-39)) on one day versus a placebo saline on another. Before surgery, blocking GLP-1 did not substantially alter brain connectivity. However, after surgery, when GLP-1 levels were high, blocking GLP-1 receptors had a notable effect: it increased connectivity in a key hub of the DMN (the lateral parietal region) compared with conditions in which GLP-1 signaling was not blocked.

In other words, when GLP-1 was allowed to act, the DMN was quieter; when GLP-1 was blocked, the DMN was activated. Furthermore, the degree of this GLP-1 effect on the DMN correlated with how much weight and appetite the patients lost - those who had the biggest GLP-1-driven changes in DMN connectivity also reported the greatest reductions in hunger and achieved larger body mass index (BMI) drops post-surgery. This suggests that GLP-1 was actively helping to tone down the brain's default activity related to appetite or self-focus, contributing to the patients' reduced desire to eat. It is a small study (N=9) and a unique scenario, but it provides a causal hint that GLP-1 signaling can quiet certain brain networks that might otherwise be promoting FRITs.

Another study by Watson et al. (2019) examined the effect of GLP-1 on the brain in a different group: middle-aged adults at risk for Alzheimer's disease (AD), many of whom had insulin resistance [11]. The focus here was on cognitive improvement, but the findings are relevant to understanding GLP-1's neural impact. In a 12-week trial, 43 participants were given either liraglutide, a GLP-1, or a placebo. Resting-state fMRI before and after treatment showed that liraglutide increased DMN connectivity over the treatment period, particularly between DMN hubs such as the medial prefrontal cortex and posterior cingulate, and the hippocampus. The placebo group did not show these changes. The authors interpreted the results as a potential strengthening or "normalization" of brain network connectivity, which might be beneficial for cognitive function. Interestingly, cognitive tests did not show improvement in that short time, but the underlying neural changes were clear in the fMRI results.

At first glance, these two studies might seem contradictory: one suggests that GLP-1 quieted a DMN region, whereas the other reports that GLP-1 increased DMN connectivity. However, it is important to note that the studies looked at different measures of connectivity. The Watson et al. study investigated connectivity between the hippocampus and the medial frontal cortex while investigating neuroprotective benefits in AD-risk patients, whereas Van Duinkerken et al. investigated right orbitofrontal cortex connectivity for its known involvement in reward processes related to food intake.

In the obesity and post-surgery scenarios, the DMN may have been overactive or engaged in food-related rumination so that GLP-1 may have dialed it down to a healthier level. In the AD-risk scenario, underlying cognitive impairments may have reduced normal DMN connectivity, so GLP-1's effect was to increase it toward normal levels. In essence, GLP-1 appears to modulate the DMN, pushing it in the direction of balance. Neither study directly measured "food noise," but both show that GLP-1s can alter the brain's default activity. This lines up with many patients' subjective reports that their mind feels "quieter" on these medications.

More research is needed to connect the dots. For instance, if we were to scan people on semaglutide while they report on their level of intrusive food thoughts, would we see a direct correlation? The existing evidence provides a strong clue: GLP-1s not only affect the gut and metabolic centers but also modulate the brain's DMN. By nudging the DMN, they might break the cycle of persistent food-related rumination.

GLP-1 and food reward: dampening the craving

So far, this review has discussed GLP-1's impact on the "background music" of the brain (the DMN). Now this review will turn to the "foreground" - how GLP-1s affect the brain's response to actual food cues and rewards.

One widely reported effect of drugs like semaglutide is a loss of interest in food. People frequently report that foods they once craved no longer call to them and that they become full more quickly and remain sated longer. This is suggestive of GLP-1 action on the brain's reward circuitry - the interconnected regions, such as the striatum, orbitofrontal cortex, amygdala, and midbrain dopamine centers, that drive the pleasure in and motivation for food. GLP-1 receptors are present in these reward regions, indicating that these drugs can directly influence the brain's wanting and liking systems [12].

Van Bloemendaal et al. (2014) conducted a pioneering human study in this area [13]. They conducted an fMRI experiment with 48 adults with obesity, in which some had type 2 diabetes, and some did not. Each participant underwent three conditions on separate days: (1) an infusion of exenatide, (2) exenatide plus a GLP-1 blocker to cancel its effect, and (3) a placebo.

While in the scanner, participants looked at pictures of high-calorie, enticing foods. The results were striking: under exenatide alone, the participants' brains showed significantly less activation in key appetite and reward regions - areas like the insula, which processes taste and internal sensations; the amygdala, which processes emotion and salience; the putamen, which processes habit and reward; and the orbitofrontal cortex, which processes valuation of rewards, were all quieter in response to the food images.

Those participants also ended up eating less at a buffet meal after the scan. However, when exenatide's action was blocked, their brain responses and eating behavior resembled those in the placebo condition. This indicates that GLP-1 signaling directly dampened both neural reactivity to food cues and actual calorie intake. In other words, with GLP-1 active, the visual allure of delicious food was muted in the brain, and people were not as motivated to eat.

Another study by van Bloemendaal and colleagues, conducted the following year, examined in greater detail how GLP-1 affects different phases of reward: wanting versus liking [2]. Participants this time were given a taste of chocolate milk while in an fMRI, but with a twist - a cue light would signal that the chocolate milk was about to be delivered through a tube, allowing the researchers to separate brain activity during anticipation (cue-triggered wanting) from activity during consumption (actual tasting pleasure). In the placebo condition, participants with obesity showed an imbalance common in overeating: their brain's reward areas lit up when the cue signaled the impending milkshake, indicating heightened craving or wanting, but their neural response during the actual tasting was blunted, indicating reduced liking.

This pattern - high anticipation, low satisfaction - may drive overeating, because people keep chasing a reward they never achieve. When the same individuals were given exenatide, this pattern shifted. Anticipatory activation in reward circuitry decreased, indicating that the cue no longer elicited as much excitement. At the same time, the brain response during consumption was normalized, suggesting a more typical pattern of heightened pleasure. They also consumed fewer calories afterward on exenatide. As in their previous study, when the GLP-1 was blocked, these effects went away. The authors noted: GLP-1 "decreases anticipatory food reward (wanting) and increases consummatory food reward (liking)." The medications lead people to want food less and to like it more.

In terms of valence (pleasure) and arousal (motivation), GLP-1 lowers the arousal or urgency triggered by food cues while boosting the positive valence of actually eating the food, thereby signaling the brain, "This is enough, you’re full."

These neuroimaging findings give a concrete mechanism for the subjective reports of reduced cravings on GLP-1 therapy. It is not willpower - the drug is literally making the brain care less about the cookies on the counter, but if you do partake, you might actually feel content after one or two instead of the whole plateful.

Animal research provides cellular-level support for these human studies. Alhadeff et al. (2012) showed that GLP-1-producing neurons in the brainstem project directly to the ventral tegmental area and nucleus accumbens - core regions of the dopamine reward pathway [12]. When they activated GLP-1 in those circuits, rodents lost interest in high-fat food; when they blocked GLP-1 there, the animals overate.

One limitation in existing human studies is that they have been conducted in people with obesity or diabetes, so we do not know if the same brain effects occur in lower BMI populations. Furthermore, many of these were short-term experiments. While there is a large and growing amount of evidence that weight loss can be maintained on GLP-1 therapy, it would be helpful to confirm with longitudinal brain scans if the effects on anticipatory food reward and consummatory food reward persist.

Nevertheless, the evidence is strong that GLP-1s blunt the brain's response to food cues and reduce craving and reward-seeking behavior. This neural perspective explains why people on these medications report feeling fewer food-related thoughts: the cues that once grabbed their brain's attention lose power, and a smaller portion of that same food feels more rewarding.

GLP-1 beyond food: addiction and cognitive effects

GLP-1s were designed to treat metabolic conditions, but as they have become widespread, benefits in seemingly unrelated areas are emerging. The ability of GLP-1s to "quiet the noise" in the reward circuit seems to extend beyond food. Two notable examples are in addiction and potentially in neurodegenerative disease.

First, consider addictions like alcohol use disorder (AUD). Like overeating, excessive drinking involves powerful cravings and a dysregulated reward system. Hendershot et al. (2025) tested semaglutide in humans with AUD [14]. In their randomized trial, 48 adults with alcohol use disorder were given either semaglutide (up to 1.0 mg weekly) or a placebo for about nine weeks. The semaglutide group experienced significantly lower self-reported alcohol cravings compared to the placebo group. In a lab session where participants had access to alcohol in an "open bar" environment, those taking semaglutide drank less - they reached lower blood alcohol levels and consumed fewer total drinks than those on placebo.

Outside the lab, semaglutide did not stop alcohol use in the study participants; in fact, they drank roughly the same number of days as before. However, they tended to drink fewer drinks per session and had more weeks without heavy drinking. Some in the semaglutide group also cut down on smoking. This was a short trial, but it suggests that GLP-1 activation can dampen the pull of other addictive substances. The mechanism is likely similar to that of food. By acting on the brain's reward centers, semaglutide made alcohol less appealing or less rewarding or both, so people were not motivated to overindulge.

The second example presents a broad overview from an observational study by Xie et al. (2025) [15]. They looked at health records of 2 million U.S. veterans and compared those who were on GLP-1s to similar patients taking other diabetes medications. The analysis covered 175 different health outcomes to see if GLP-1 use was associated with any patterns beyond blood sugar control. The findings were striking. People taking GLP-1s had a lower incidence of a range of conditions, including fewer new cases of substance use disorders, fewer anxiety and depression diagnoses, and fewer diagnoses of neurocognitive disorders such as dementia. They also observed benefits such as lower rates of heart disease and all-cause mortality, but for the purposes of this review, the mental health and brain-related outcomes stand out. The dementia finding is consistent with research suggesting that GLP-1 may have neuroprotective effects. While this was not a randomized controlled trial and cannot prove causation, it is suggestive.

Together, these studies suggest that what the GLP-1 does for food-related noise, it may also do in other domains, such as drug addiction. We also see hints of psychological benefits - people on a GLP-1 may feel less anxious or depressed, which could be related to breaking vicious cycles, like stress->eat->stress.

What is clear is that GLP-1 effects extend beyond the desire for food. GLP-1s affect the reward and motivation systems of the brain in a foundational way, recalibrating the brain toward a calmer state where temptations are less loud and, perhaps, where one's cognitive processes are healthier. The findings reinforce the idea that combining GLP-1 with behavioral or psychological strategies could address both the physiological and psychological aspects of cravings, which brings us to mindfulness and the role of mindful eating.

Mindfulness, rumination, and the DMN

Mindfulness-based practices, especially mindfulness meditation, have gained traction as tools to manage overeating and cravings. Mindful eating programs, such as Noom's, encourage individuals to slow down and pay attention to the eating experience: notice the colors and smells of food, savor each bite, and tune in to the body’s hunger and satiety signals.

Equally important, mindfulness teaches awareness of thoughts and feelings in a nonjudgmental way. For someone struggling with "food noise," this means learning to notice the mind-wandering thought "I want chocolate now" as just a thought, not a mandate.

Like GLP-1s, mindfulness affects the brain's DMN. A study by Brewer et al. (2011) compared mindfulness meditators, who had thousands of hours of practice, to non-meditators during fMRI scans [16]. The meditators were scanned while they engaged in meditation and during a normal resting state. The results showed that when the experienced meditators were meditating, they had significantly lower activation in the core DMN hubs - the medial prefrontal cortex and posterior cingulate cortex - compared to the non-meditators.

Even at rest, long-term meditators showed, on average, less default mode activity than the control group. The meditators' brains also displayed stronger connectivity between the default mode regions and parts of the executive control network. This likely reflects either the ability to monitor and suppress mind-wandering or the natural executive-function gains that occur when mind-wandering is suppressed.

In essence, the study demonstrated that experienced meditators can turn down the volume on the DMN. Brewer et al. noted that these neural findings correlated with the meditators' own reports of feeling less caught up in their thoughts day to day [15]. This study suggests why mindfulness might help with conditions involving rumination - the practice changes the brain’s connectivity so that the usual default mode chatter is quieter.

The link between mindfulness, DMN, and rumination is illustrated in the context of depression, where rumination is a fundamental issue. Depressive rumination involves getting stuck in negative, self-focused thought loops, often about the future. Van der Velden et al. (2023) conducted a randomized trial of Mindfulness-Based Cognitive Therapy (MBCT) for people with a history of depression, in which participants were scanned before and after an eight-week mindfulness training versus a control group with no training [17].

During the scans, the investigators induced a self-critical rumination state by having participants think about personal failures. They also had participants focus only on their breath. After the training, the MBCT group showed a notable change: during the induced rumination, connectivity decreased between the salience network, which toggles between the default mode and focused attention, and the visual memory regions that were fueling the rumination.

In other words, mindfulness training disrupted the pattern of getting "stuck" in a negative thought loop. At the same time, the participants learned to redirect attention to bodily sensations, such as the breath, and their scans showed that they could better engage regions involved in present-moment attention. Consistent with this, they reported less tendency to ruminate and had lower depression scores after the training than the control group. This study shows that practicing mindfulness can rewire how different brain networks interact when one starts to spiral into rumination: the training strengthened the ability to quiet the default mode.

A central mechanism through which mindfulness-based interventions reduce overeating is by interrupting automatic cue-response patterns. Instead of feeling stress and automatically reaching for a snack, mindfulness adds a pause. You become aware, "I’m feeling stressed and I want chocolate." You acknowledge the thought or craving without immediately acting on it. You might take a few deep breaths and notice the craving pass, or make a conscious choice to eat something more nourishing, or decide to eat the chocolate.

Neurologically, this corresponds to activating executive control regions and disengaging the default-mode rumination that would normally lead to the treat. Over time, this practice can significantly reduce the power of cravings. By dampening the DMN and enhancing conscious control, mindfulness quiets food noise. While not everyone finds mindfulness practical, the changes in both brain and behavior can be profound with practice.

**Figure 2:**
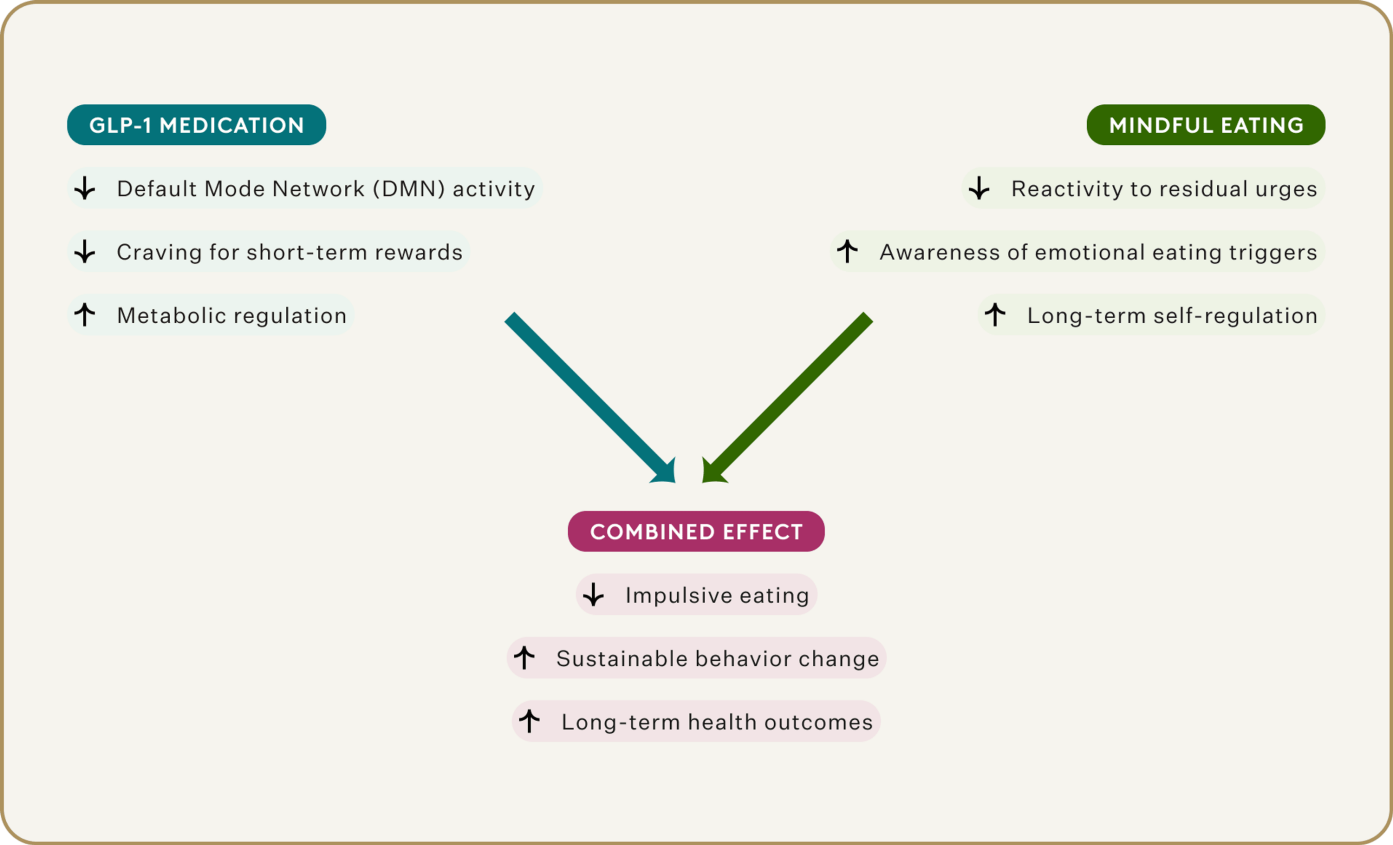
Dual Intervention Model Permission was obtained from the original publisher [18].

We have seen, with GLP-1 and now with mindfulness, that both reduce rumination and "food noise," but through very different mechanisms, raising a central question: could they be complementary rather than alternative interventions? (Figure 2).

GLP-1 and mindfulness: two paths to a quieter mind

Having looked at GLP-1s and mindfulness separately, it is striking how they converge to quiet the DMN and silence "food noise." Both help people eat, in a sense, more mindfully: GLP-1 by chemically reducing hunger and cravings and modulating rewards to increase liking and decrease wanting, and mindfulness by training the mind to eat less impulsively, perhaps also decreasing wanting via rational thought and increasing liking via techniques like savoring.

Both GLP-1 and mindfulness lead to eating less and to fewer cravings. Neuroimaging indicates that each can reduce activity in the insula, a region that integrates bodily hunger signals and reward-related cravings. With GLP-1, the insula's response to tempting food cues drops [2].

Beyond food, GLP-1 users report less interest in alcohol or other compulsive behaviors [14,15]. Mindfulness practitioners often find that, as they become more aware and less reactive, habits such as stress-eating or smoking can diminish. Both can positively influence mood and stress levels: GLP-1 through direct effects on the brain, and mindfulness through improved emotional regulation.

Despite these parallels, the mechanisms of action may be opposite. GLP-1 is a biological intervention that requires no patient effort beyond taking the medication. Mindfulness, meanwhile, is psychological - a trained capacity to alter responses to cravings rather than preventing them from arising. When practiced, mindfulness may also prevent these cravings from arising in the first place. In short, GLP-1 blunts the signal; mindfulness changes how the signal is handled when it appears, yet both lead to the same result: reduced activity in the brain’s reward centers and less food noise.

Consequently, each has its limitations. GLP-1 can cause side effects, can be expensive, and, upon medication discontinuation, absent underlying behavioral change, the appetite suppression may resolve. Mindfulness, by contrast, is safe and inexpensive, and the skills acquired can last a lifetime.

There is reason to believe one could complement the other. People taking GLP-1s may have a window during which food-related noise is quited and motivation for change is unusually high. The vast majority of individuals seeking to lose weight with weight-loss medications believe they will also adopt healthier behaviors [19]. GLP-1s reliably deliver weight loss - the open question is whether that pharmacologic "quiet" can be leveraged for durable behavior change during the window when change is easiest.

Once the intrusive chatter is quieted, intention is no longer competing with rumination. That may be the perfect moment to introduce mindfulness, healthy eating, and movement habits. When medication and healthy habits are adopted simultaneously, success on the scale amplifies motivation, which in turn fuels further action. Action fuels motivation. Health becomes a habit. This is how health journeys deepen: a diet becomes a half-marathon, which becomes a triathlon.

And critically, when the medication is eventually discontinued - as it is for the vast majority - the individual is not returned to baseline. They separate from the drug with a new set of psychological habits and behaviors in place, reducing regression and preserving gains.

Because formal studies on combining GLP-1 at varying dosages with mindful eating practices are lacking so far, research in this area is needed. GLP-1 may reduce the "pull" - the positive arousal - of food cues, and mindfulness may help one reframe the "push," deciding not to pursue the cue because their identity has changed to someone who generally makes the healthy choice. Mindfulness could reinforce the GLP-1 and the GLP-1 the mindfulness.

Another difference worth noting is how each affects the DMN. GLP-1's effect on the default mode may be a side effect of improved metabolic signaling - or it may be more profound than that. Meanwhile, mindfulness's effect on the DMN is direct: it is a practice specifically aimed at noticing and letting go of wandering thoughts, thereby deactivating the DMN. It follows that mindfulness may be better at addressing types of food noise that are more cognitive or emotion-driven, like "stress eating," whereas GLP-1 will modulate the reward networks to make eating past satiety less likely. For this reason, a combined approach may be sensible.

## Conclusions

The evidence reviewed in this paper suggests that food noise may be understood as a form of maladaptive prospection - a cognitive process involving repetitive, cue-elicited simulations of short-term reward that can interfere with longer-term goals. Emerging findings indicate that GLP-1 receptor agonists may influence neural and cognitive mechanisms underlying this phenomenon, including reward anticipation and cue reactivity, whereas mindfulness-based interventions appear to act on attentional regulation and aspects of DMN activity relevant to craving and rumination. Although both approaches show potential to modulate processes related to intrusive food-related thought, the existing data remain preliminary, indirect, and derived primarily from small, heterogeneous studies. Current evidence does not establish causal pathways linking GLP-1 therapy or mindfulness practice to reductions in food noise, nor does it demonstrate that either intervention produces downstream behavioral changes through the mechanisms proposed here. Thus, the conceptual model offered in this review - linking reductions in maladaptive prospection to shifts across DMN, salience, and executive networks - should be regarded as a hypothesis-generating framework rather than a confirmed explanation. No meta-analysis was undertaken in this review, and findings are based on a qualitative synthesis of selected studies.

Future research should directly measure food noise, employ longitudinal and neuroimaging methods, and test whether GLP-1 therapy, mindfulness training, or their combination produces distinct or synergistic effects on prospection, craving, and eating behavior across diverse populations. Taken together, current findings provide an initial basis for investigating how biological and psychological interventions may converge on shared cognitive processes involved in craving and prospective thought. Substantial empirical work is still needed to validate these mechanisms, determine causality, and assess their relevance for clinical practice.
